# Development and validation of nomogram combining serum biomarker for predicting survival in patients with resected rectal cancer

**DOI:** 10.1042/BSR20192636

**Published:** 2019-11-13

**Authors:** Shaonan Fan, Ting Li, Ping Zhou, Qiliang Peng, Yaqun Zhu

**Affiliations:** Department of Radiation Oncology, Second Affiliated Hospital of Soochow University, Suzhou 215004, China

**Keywords:** CEA, colorectal cancer, Nomogram, overall survival

## Abstract

Purpose: Nomogram is a widely used tool that precisely predicts individualized cancer prognoses. We aimed to develop and validate a reliable nomogram including serum tumor biomarkers to predict individual overall survival (OS) for patients with resected rectal cancer (RC) and compare the predictive value with the American Joint Committee on Cancer (AJCC) stages.

Patients and methods: We analyzed 520 patients who were diagnosed with non-metastatic rectal cancer as training cohort. External validation was performed in a cohort of 11851 patients from the Surveillance, Epidemiology, and End Results (SEER) database. Independent prognostic factors were identified and integrated to build a nomogram using the Cox proportional hazard regression model. The nomogram was evaluated by Harrell’s concordance index (C-index) and calibration plots in both training and validation cohort.

Results: The calibration curves for probability of 1-, 3-, and 5-year OS in both cohorts showed favorable accordance between the nomogram prediction and the actual observation. The C-indices of the nomograms to predict OS were 0.71 in training cohort and 0.69 in the SEER cohort, which were higher than that of the seventh edition American Joint Committee on Cancer TNM staging system for predicting OS (training cohort, 0.71 vs. 0.58, respectively; *P*-value < 0.001; validation cohort, 0.69 vs. 0.57, respectively; *P*-value < 0.001).

Conclusion: We developed and validated a novel nomogram based on CEA and other factors for predicting OS in patients with resected RC, which could assist clinical decision making and improvement of prognosis prediction for individual RC patients after surgery.

## Introduction

Colorectal cancer (CRC) is the fourth most common cancer and ranks second in terms of mortality in the United States [1]. In 2018, it is estimated that more than 1.8 million new cases of colorectal cancer and 881,000 deaths occurred in the world, accounting for approximately 10% cancer cases and deaths [2]. According to the latest data released by the National Cancer Center, there were 370,000 new cases of colorectal cancer in China in 2015, accounting for 26% of the world. The incidence and mortality were 28 and 13.61 per 100,000, ranking third and fifth among all malignant tumors, respectively [3]. Favorable long-term survival and a better quality of life after a curative resection are usually achieved in patients with non-metastatic rectal cancer (RC) [4]; however, distant metastases and local recurrences that result in poor survival still remain the primary concerns in this entity [5].

The tumor, node, metastasis (TNM) staging system is a widely used cancer staging system for predicting the prognosis and determining the appropriate treatment strategy. In the seventh edition of the American Joint Committee on Cancer TNM classification, patients with non-metastatic rectal cancer (RC) are stratified according to the depth of invasion and extent of lymph node involvement [6]. Although the TNM stage is important and useful, it is well known that the survival time varies widely, even in patients with the same stage of disease [7]. What convinced is that there are other independent prognostic factors such as sex, age, histology, and treatment-related factors could be significantly devoted to individualized survival prediction [6]. Recently, serum biomarkers have been widely used in clinical diagnosis, post-operative monitoring and prognosis of CRC patients [8]. Common biomarkers for RC including carcinoembryonic antigen (CEA) and tumor-associated antigens, such as cancer antigen 19-9 (CA19-9), cancer antigen 50 (CA50), and cancer antigen 72-4 (CA72-4), whose predictive significance of pre-operative levels in different tumor populations has been extensively documented in numerous studies [9–12].

Nomogram is an intuitive graph of a statistical predictive model that generates a numerical probability of a clinical event, such as overall survival (OS) by incorporating important factors for oncologic prognosis, and it has been widely practiced in clinical application [13,14]. Comparing with the traditional TNM staging systems or other staging systems, the nomogram has been proved with the precise predictive value in other types of tumors [14–16]. However, a reliable nomogram based on common serum tumor biomarkers and other clinicopathologic index for predicting long-term survival outcome of patients with resected RC was scarce.

Therefore, the aim of this study was to develop a nomogram for individual post-operative prediction in patients with RC by combining known clinicopathologic variables (including tumor biomarkers) based on the data from a single-institutional registry in China. In addition, we used a separate cohort from the Surveillance, Epidemiology, and End Results (SEER) database to externally validate it [17].

## Methods and materials

### Patient selection

For the SEER cohort, by using the SEER*Stat, version 8.3.5 software, we selected RC patients diagnosed between 2004 and 2015 [18]. The inclusion criteria for selected patients were as follows: (1) Patients aged 18 years or older; (2) patients with RC diagnosed as the first primary cancer; (3) patients with RC underwent surgical resection without neoadjuvant therapy; (4) the International Classification of Diseases for Oncology third edition (ICD-O-3) codes (8010-8231 and 8255-8576); and (5) patients who were followed up for at least 3 months after surgery were included in the study. In the training cohort, patients with RC received surgery between January 2008 and December 2015 in the Second Affiliated Hospital of Soochow University were included and following inclusion criteria: Patients aged 18 years or older; underwent surgical resection; patients who were followed up for at least 3 months after surgery were included in the study; no synchronous distant metastases or other malignancies; no neoadjuvant therapy; serum biomarker levels within 6 months of surgery were available. The same variables used were age at diagnosis, gender, T stage, N stage, histological subtypes, histological grade, surgical margin, tumor deposits, and the levels of preoperative CEA.

### Statistical analysis

Continuous variables were compared using the Student’s *t*-test (normally distributed data) or the Mann–Whitney *U-*test (non-normal distribution data), as appropriate. Categorical variables were compared using the Chi-square test. The primary endpoint of this study was overall survival (OS), which was defined as the interval between the initial diagnosis of RC and the last follow-up. The Kaplan–Meier method was used to estimate the survival probabilities and the log-rank test was used to assess the survival differences of OS. Univariate and multivariate Cox regression hazards models were employed to identify prognostic factors. The development of the final nomogram was based on the prognostic factors extracted from the training cohort and validation of the nomogram was carried out using the data from the SEER cohort. The validation of the nomogram was performed using the concordance index (C-index), calibration curves. Finally, a risk classification system was established according to the total scores of each RC patient in the training cohort by using the nomogram to separate patients into three prognostic groups, i.e. the low-, intermediate-, and high-risk groups. The X-tile (Yale University, 3.6.1) was implemented to calculate cut-off points for prognostic groups. The risk classification system was further validated in the SEER cohort. All statistical analyses were completed using R (version 3.5.2, R Foundation for Statistical Computing, Vienna, Austria). All statistical tests were two-sided and statistical significance was set at *P*-value < 0.05.

## Results

### Clinical characteristics

The baseline characteristics of the training and validation cohort were shown in Table 1. All patients received surgery. In training cohort, a total of 158 (30.4%) patients had positive preoperative CEA (≥6.5 ng/ml) and 38 (7.3%) patients had positive preoperative CA19-9 (≥37.0 U/ml). Post-operative pathological tumor staging suggested that 24 patients were in the T1 stage, 150 patients in the T2 stage, 272 in the T3 stage, and 74 were in the T4 stage, while 348 patients were in the N0 stage, 116 in the N1 stage, and 56 in the N2 stage. The positive rates of vascular invasion and perineural involvement were 18.1% (94/520) and 13.1% (68/520), respectively. For both training and validation cohorts, the majority of patients were with T3 and N0 stages and the most common histological subtype was adenocarcinoma. Ignoring the absence of certain parameters (e.g. surgical type, vascular invasion, perineural involvement, and preoperative CA19-9 level) in the validation cohort, there were no significant differences in the clinical feature characteristics between the training and the validation cohort, which justified their values as training and validation cohorts.

**Table 1:** Baseline characteristics of RC patients in the training and SEER cohort

Patient characteristics		Training cohort (*n*=520)	SEER cohort (*n*=11,851)	*P*-value
Age (mean (SD))		62.86 (11.17)	60.91 (12.65)	0.001
Gender (%)	Female	200 (38.5)	4682 (39.5)	0.666
	Male	320 (61.5)	7169 (60.5)	
Surgical type (%)	Dixon	330 (63.5)	–	
	Others	190 (36.5)	–	
T stage (%)	T1	24 (4.6)	1622 (13.7)	<0.001
	T2	150 (28.8)	2323 (19.6)	
	T3	272 (52.3)	7002 (59.1)	
	T4	74 (14.2)	904 (7.6)	
N stage (%)	N0	348 (66.9)	6469 (54.6)	<0.001
	N1	116 (22.3)	4180 (35.3)	
	N2	56 (10.8)	1202 (10.1)	
Histopathology (%)	Adenocarcinoma	488 (93.8)	11194 (94.5)	0.62
	Others	32 (6.2)	657(5.5)	
Grade (%)	G1/G2	476 (91.5)	10369 (87.5)	<0.001
	G3/G4	26 (5.0)	1482 (12.5)	
	NR	18 (3.5)	–	
Vascular invasion (%)	No	426 (81.9)	–	
	Yes	94 (18.1)	–	
Perineural involvement (%)	No	452 (86.9)	–	
	Yes	68 (13.1)	–	
Margin (%)	No	518 (99.6)	7071 (59.7)	<0.001
	Yes	2 (0.4)	532 (4.5)	
	NR	–	4248 (35.8)	
Tumor Deposits (%)	No	450 (86.5)	7451 (62.9)	<0.001
	NR	10 (1.9)	3781 (31.9)	
	Yes	60 (11.5)	619 (5.2)	
Preoperative CEA (%)	Negative	362 (69.6)	7534 (63.6)	0.006
	Postive	158 (30.4)	4317 (36.4)	
Preoperative CA199 (%)	Negative	482 (92.7)	–	
	Postive	38 (7.3)	–	

Abbreviations: RC, rectal cancer; SD, Standard Deviation; SEER, Surveillance, Epidemiology and End Results; NR, not recorded.

### Univariate and multivariate Cox regression of the training cohort

**Figure 1:**
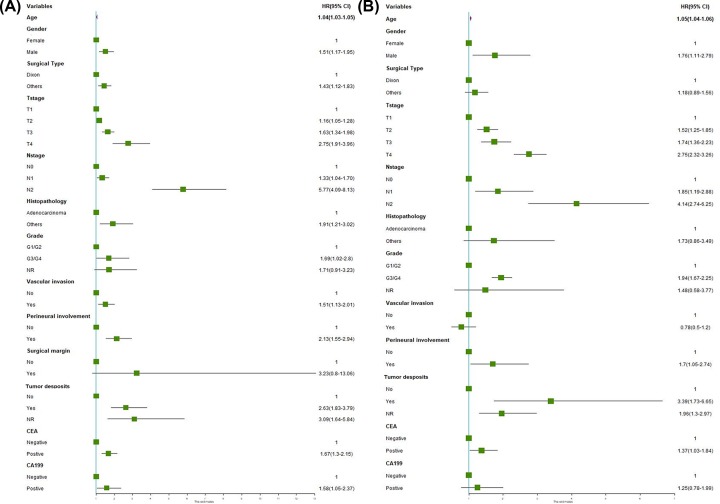
Analysis of clinicopathological information with OS in the training cohort (**A**) Univariate and (**B**) multivariate analysis of clinicopathological information with OS. Abbreviations: OS, over survival; NR, not recorded; HR: hazard ratio; 95%CI: 95% confidence interval.

For the training cohort, univariate analyses were performed to identify clinical variables that were significantly associated with OS. As shown in Figure 1A, female sex and younger age were associated with better prognosis (*P*-value < 0.05). With respect to factors associated with surgery, we found that pathologic T and N stages, tumor differentiation, histological subtype, vascular invasion, perineural involvement (PNI), and tumor deposits (TDs) status were greatly associated with OS (*P*-value < 0.05). In addition, the negative correlation of preoperative CEA (HR: 1.67 95% CI: 1.3–2.15) and CA19-9 (HR: 1.58; 95% CI: 1.05–2.37) levels with OS were significant (*P*-value < 0.05). After univariable analysis in the training cohort, the variables that achieved significance (*P*-value < 0.05) were factored into multivariable analysis in the Cox proportional hazard regression model. Finally, only eight factors (age, sex, grade, PNI, TDs, preoperative CEA, T stage and N stage) remained independent prognostic factors for overall survival (Figure 1B).

### Prognostic nomogram for OS

**Figure 2:**
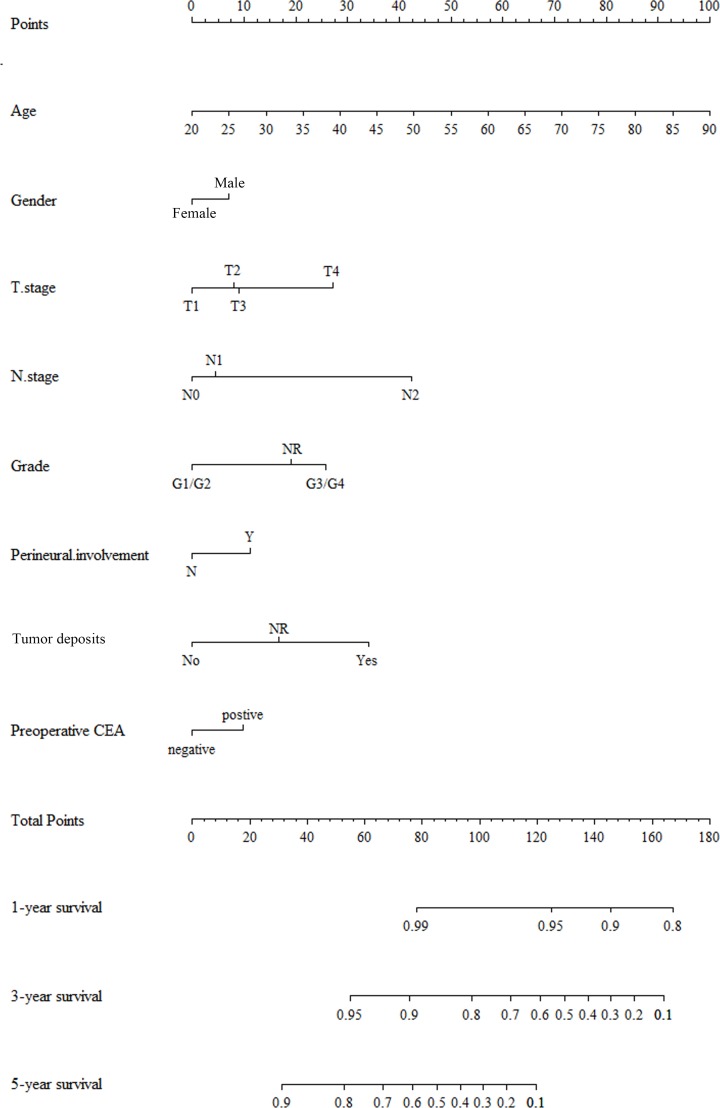
Nomogram integrating tumor markers and clinicopathological factors

Nomogram for predicting 1-, 3-, and 5-year OS was constructed by incorporating the above independent prognostic factors (Figure 2). The nomogram illustrated age and N stage as sharing the largest contribution to prognosis, followed by the T category and TDs status. Each subtype within these variables was assigned a score on the point scale. A straight line can be easily drawn down by adding the scores of all the selected variables to determine the survival probabilities of the individual patients.

### Validation of the nomograms

**Figure 3:**
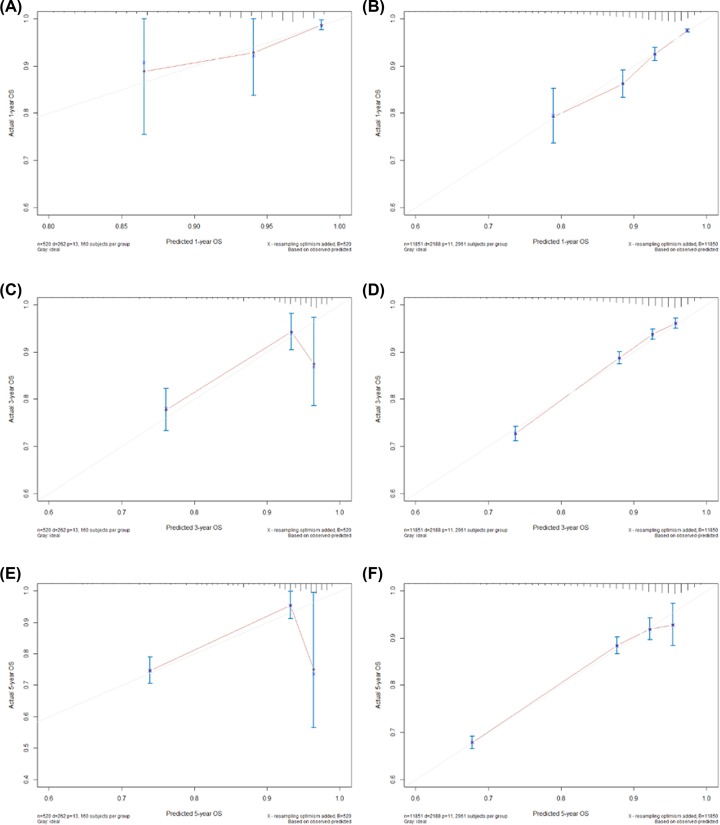
Evaluation of nomogram using calibration curves 1-, 3-, and 5-year nomogram calibration curves in the training cohort (**A,C,E**) and SEER cohort (**B,D,F**). The dashed line represents an ideal evaluation, whereas the red line represents the performance of the nomogram.

The C-indices of the nomogram to predict OS were 0.71 (95%CI:0.64–0.79) in training cohort and 0.69 (95%CI:0.61–0.78) in validation cohort. The calibration plots presented an acceptable agreement in the training cohort and an excellent agreement in SEER validation cohort between the nomogram prediction and actual observation for 1-, 3-, and 5-year OS (Figure 3). We further compared the OS predictive ability between the nomogram and TNM staging system. In the training cohort, the Harrell’s C-index for the established nomogram to predict OS (0.71; 95%CI: 0.64–0.79) was significantly higher than that of the TNM staging system (0.58; 95%CI: 0.54–0.62; *P*-value < 0.001). In the SEER cohort, the difference is also statistically significant (0.69 vs. 0.57; *P*-value < 0.001).

### Performance of the nomogram in stratifying risk of patients

**Figure 4:**
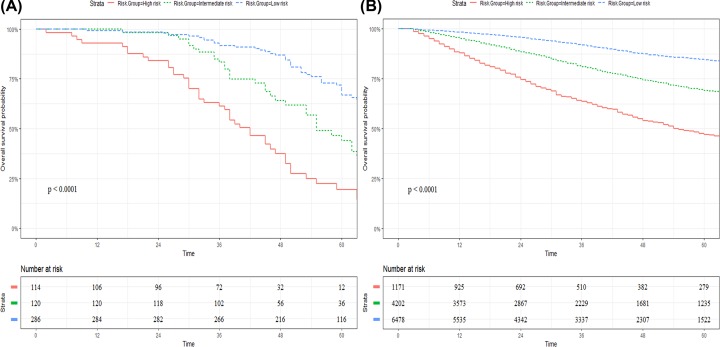
Kaplan–Meier curves of OS for patients in the low-, intermediate-, and high-risk groups in the training cohort (**A**) and SEER cohort (**B**)

Based on the quartiles of the nomogram score from the training cohort, we determined the cut-off values by grouping the patients in both cohorts evenly into three proposed risk groups; each group represented a distinct prognosis. As shown in Figure 4, the subgroups experienced distinct overall survival in the training cohort and the SEER cohort (*P*-value < 0.001).

### Comparisons of blood biomarkers in pre- and post-operative

**Figure 5:**
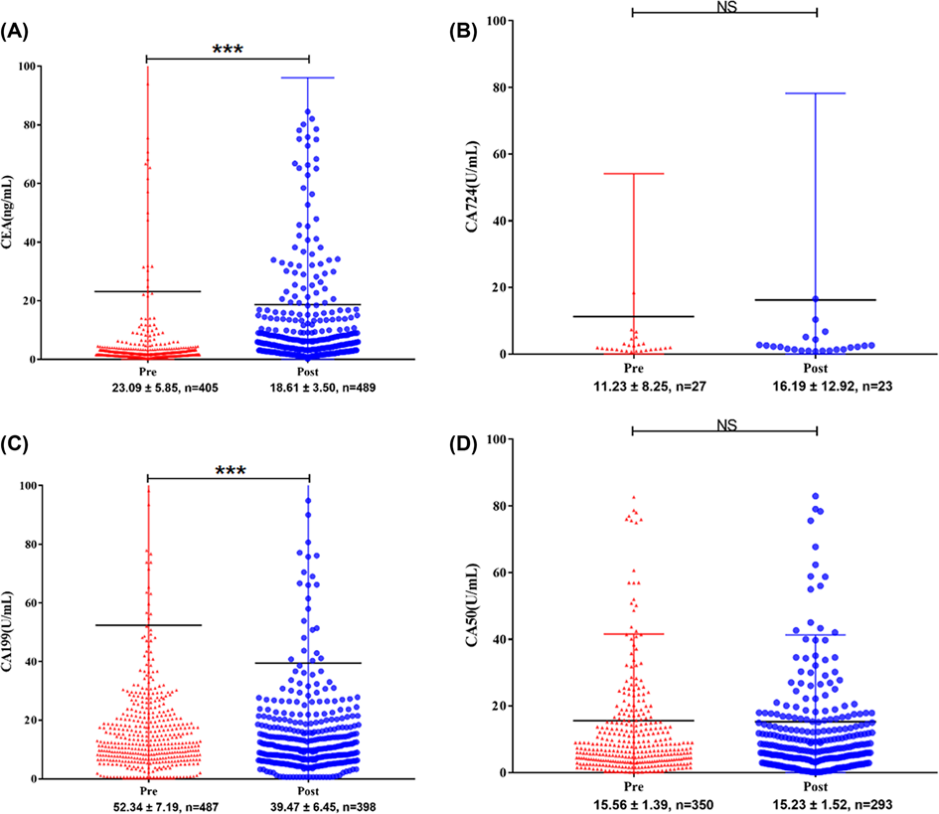
Distribution of serum biomarkers, including Carcinoembryonic antigen (CEA) (**A**), Cancer antigen 724 (CA724) (**B**), Cancer antigen 199 (CA199) (**C**) and Cancer antigen 50 (CA50) (**D**) in rectal cancer patients pre- and postoperative Abbreviations: NS, no significance; ***, *P*-value < 0.001

Our results indicated the profile of CEA and CA19-9 undergone profound changes before and after surgery in RC patients. The mean values of the pre-operative CEA and CA19-9 level were 23.09 ± 5.85 ng/ml and 52.34 ± 7.19 U/ml, respectively. However, the changes in pre-operative and post-operative CA50 levels (pre-post-CA50) were not statistically significant. Due to the limitation of the number of cases, the changes in the levels of pre- and post-operative CA72-4 remain ambiguous (Figure 5).

## Discussion

Due to the heterogeneity of RC in the survival of individual patients, the TNM staging system for predicting survival is inaccurate and insufficient to satisfy the medical demand [4]. Based on multivariate analysis to identify proper risk factors that are significantly associated with decreased survival, the nomogram could help clinicians to select patients with high risk of recurrence and metastasis, obtain better personalized prognostic assessments in clinical practice, which may be better than traditional TNM staging system in several cancers [19]. In current research, our multivariate analysis revealed that several factors are independent negative predictive determinants for OS (Figure 1B). Thus, we strive to develop a postoperative nomogram to predict long-term survival of patients with resected RC.

Tumor biomarkers, substances expressed and released during tumorigenesis and progression, give information about tumor biology in an indirect way and may reveal the presence of a new growth that indicates the relative tumor burden and biological aggressiveness [20]. For example, disruption of normal tissue structure and loss of polarity of tumor cells leads to CEA expression on the entire cell surface, which is then secreted into the bloodstream, ultimately increasing serum CEA levels. Because of its convenience and cost-effectiveness, tumor biomarkers are expected to guide therapy schedules and monitor prognosis [21]. Although CEA is not a disease-specific marker and National Comprehensive Cancer Network guidelines also did not include elevated serum CEA levels as a high-risk factor, recent studies, as well as our findings support its effectiveness for monitoring or evaluating treatment in RC patients. Moreover, changes between the preoperative and postoperative CEA levels are also suggested as indicators of higher recurrence rate.

Undoubtedly, the suggested cut-off values of preoperative CEA values were different, ranging from 2.5 to 50 ng/ml [22–24]. Generally, the cut-off level of 5 ng/ml was used to define CEA elevation. However, in our study, in order to make the nomogram better used in clinical practice of our institution, we defined CEA ≥ 6.5 ng/ml as positive in the training cohort, which is based on the reference value provided by the clinical testing center of our institution.

In the present study, we performed a retrospective study in patients with resected RC who had four tumor biomarkers (CEA, CA19-9, CA50 and CA724) examined preoperatively. However, the number of cases of CA724 and CA50 in the training cohort was relatively insufficient, especially preoperative CA724, which was only effectively recorded in 27 cases (Figure 5), less than 10%. Thus, in the subsequent univariate analysis, we did not include it in the scope of the analysis. In addition, information of these two serum markers is not available in the open-accessed SEER database. Our results indicated the profile of CEA and CA19-9 undergone profound changes before and after surgery in RC patients. Through univariable analysis and subsequent multivariable analysis, we identified age, sex, grade, perineural involvement, tumor deposits, preoperative CEA, T stage and N stage as independent prognostic factors (Figure 1B). These findings were in high concordance with previous reports on risk factors for RC [25,26].

It is essential to verify the nomogram in order to avoid over-fitting of the matrix and determine its universality [27]. We externally validated the nomogram using an independent cohort from the SEER database. In the present study, the calibration plot showed a first-rank consistency between the predicted and actual observation, supporting the reproducibility and reliability of the created nomogram (Figure 3). The C-index of the nomogram achieved 0.71 (95%CI:0.64–0.79) in the training cohort and 0.69 (95%CI:0.61–0.78) in the SEER cohort, both of which were comparable to those of other nomograms for rectal cancer [4,13]. Compared with the TNM staging system in the training group, the C-index of the nomogram is significantly higher, thus revealing discrimination and the discriminative ability was only slightly reduced in validation cohort. In addition, Kaplan–Meier estimates of the event rates over time for the validation cohort showed statistically different outcomes for the three proposed risk groups, which displayed a favorable discrimination and generalizability. More importantly, the model also fit the SEER cohort, which supports multi-country use of this nomogram, regardless of race and health care disparities.

Previous studies have established nomograms containing serum markers such as CEA to predict the prognosis for CRC patients. Zhang et al. [28] selected eligible CRC patients from the SEER database and created a nomogram containing CEA expressions and other clinic-pathological factors, which showed accuracy and risk assessment ability than the AJCC staging system. Although this study is based on a large number of real-world populations, the SEER database records information on malignant tumors in the United States, and prognosis of colorectal cancer varies among different populations. Therefore, whether it is universal or not requires further verification. Another eastern-population based retrospective study have comprehensively considered the expression of CEA, CA50, and CA724 to assess their clinical significances in predicting prognosis and built nomograms to predict the overall survival (OS) and disease-free survival (DFS) in CRC patients after radical resection, indicating the above tumor biomarkers have an important role in monitoring recurrence and metastasis [4]. However, as a retrospective study, the limitations caused by the sample size are inevitable. Therefore, their nomograms may represent a better preferential ability to improve prognosis prediction for CRC patients, If could verify by using a large population-based approach with longer durations of follow-up. Compared to previous studies, our study has established a nomogram based on the real-world data. This nomogram contains not only preoperative CEA but also other clinical pathologic variables (age, sex, grade, PNI, TDs, T, and N Stage). In addition, we conducted external validation by using the SEER database, which was mainly derived from the western population and increased the reliability of the nomogram**.**

We acknowledged several limitations in our study. First, our study was depended exclusively on a single institutional retrospective analysis, although eligibility criteria have been developed to minimize the selective bias, and we failed to incorporate some important molecular factors (e.g. KRAS, NRAS, BRAF mutation, MSI, or MMR). Second, the information of PNI was unavailable in the SEER cohort, we excluded this parameter during verification, which may limit the power of external validation. In addition, detailed information on recurrence, performance status and postoperative treatment, especially the pharmacological treatments (neoadjuvant/ adjuvant/ others) received by the patients, which are not available in the open-accessed SEER database, was not evaluated in our study.

At present, neoadjuvant therapy for rectal cancer has become the standard treatment strategy for locally advanced rectal cancer (cT3-4, or N+) patients. The complete pathological response after neoadjuvant therapy may be the most reliable prognostic factor, and building a nomogram containing the T downstaging after neo-adjuvant treatment may better predict the survival of patients with rectal cancer. However, limited by the sample size, the patients with neoadjuvant therapy were excluded in our study.

Despite the above-mentioned limitations, we established and validated a novel nomogram for predicting survival of patients with resected RC. To the best of our knowledge, this is the first nomogram for predicting survival of patients with resected RC that is based on a single institutional database with long-term follow-up and validated by SEER database. This model could help physicians consider preoperation serum levels of CEA and other factors to more precisely estimate the survival of individual patients after surgery, and identify those high-risk patients and support decision-making in clinical practice and follow-up strategies. Further studies are warranted on prospective, multi-institutional, large sample size data collection and individualized follow-up, broader geographic recruitment, and incorporation of some other factors (e.g. important molecular factors, the pharmacological treatments, the complete pathological response and T downstaging after neo-adjuvant treatment, etc.) to validate and improve this model in the treatment decision-making field of rectal cancer.
